# High-density Lipoprotein Cholesterol, Cognitive Function and Mortality in a U.S. National Cohort

**DOI:** 10.1186/1476-511X-10-26

**Published:** 2011-01-28

**Authors:** Richard F Gillum, Thomas O Obisesan

**Affiliations:** 1 Department of Medicine Howard University College of Medicine Washington, DC, USA

## Abstract

Low levels of both high density lipoprotein cholesterol (HDL) and cognitive function are associated with increased mortality risk. HDL plays an important role in brain metabolism. We test the hypotheses that the relative protective effect of high HDL level as related to mortality is greater in persons with impaired cognitive function than in others. Data were analyzed from a longitudinal mortality follow-up study of 4911 American men and women aged 60 years and over examined in 1988-1994 followed an average 8.5 yr. Measurements at baseline included HDL, a short index of cognitive function (SICF), socio-demographics, health status, and self-reported leisure-time physical activity. In proportional hazards regression analysis, no significant interaction of HDL with cognitive function was found (p = 0.08); there was a significant age-SICF interaction. After stratifying by age and adjusting for confounding by multiple variables, independent associations of HDL and SICF score with survival were strongest among the oldest persons. Consistent with its association with HDL, cognitive function and survival, controlling in addition for physical activity reduced the associations. In a nationwide cohort of older Americans, analyses demonstrated a lower risk of death independent of confounders among those high HDL and SICF scores, strongest among the oldest persons.

## Findings

Both low high-density lipoprotein cholesterol (HDL), and impaired cognitive function are prevalent concomitants of aging in industrialized nations [1,2]. Studies of HDL and cognitive function and Alzheimer's disease suggest a possible negative association [3]. Recently HDL has been found to play important roles in brain pathophysiology [4-6]. Cognitive function has been found to predict subsequent mortality in elderly adults in a number of previous studies [7]. Mechanisms remain obscure. High HDL has been found to be negatively related to increased mortality from cardiovascular disease [8,9]. Mechanisms may include tissue cholesterol removal, fibrinolytic activity, hemostatic function, inflammation and oxidative stress leading to less atherosclerosis. However, information regarding interactions of cognitive function and HDL cholesterol are lacking in studies of mortality.

We test the hypothesis that the effect of HDL on mortality is modified by cognitive function score, the effect being less among those with high compared to those with low scores. We analyzed available data from a national health examination survey linked-mortality file conducted with scientific sampling and state-of-the-art interviewing, examination and laboratory methods.

The Third National Health and Nutrition Examination Survey (NHANES III) was conducted in 1988-1994 on a nationwide multi-stage probability sample of 39,695 persons from the civilian, non-institutionalized population aged 2 months and over of the United States. Persons aged 60 and over, African Americans, and Mexican Americans were oversampled. Details of the plan, sampling, operation, response and institutional review board approval have been published as have procedures used to obtain informed consent and to maintain confidentiality of information obtained [10]. The personal interviews and physical and laboratory examinations of NHANES III subjects provided the baseline data for the study. This analysis was based on the public-use version of the NHANES III linked-mortality file with mortality data through 2000. Of 33,994 persons with baseline interview data, 13,944 were under age 17 and 26 lacked data for matching leaving 20,024 eligible for mortality follow-up. The NHANES III linked mortality file contains information based upon the results from a probabilistic match between NHANES III and the NCHS National Death Index records. The NHANES III linked mortality file provides mortality follow-up data from the date of NHANES III survey participation (1988-1994) through December 31, 2000.

Of the 20,022 interviewed persons with mortality follow-up, 6588 were aged 60 years and over and eligible to have cognitive function testing performed, 6339 of whom had valid cognitive function data. After excluding persons with missing data for any of the variables shown in the tables, 4,911 persons aged 60 and over with complete data remained for mortality analyses. The length of follow-up of survivors ranged from 75 to 146 months, mean 108 months, median 107 months.

HDL. Serum lipids were measured at the Centers for Disease Control and Prevention in Atlanta, Georgia. HDL was measured in serum following the precipitation of other lipoproteins with a polyanion/divalent cation mixture. For descriptive analysis, quartiles of HDL concentration were formed. For regression analysis, persons with concentrations below 40 mg/dL were compared to those with 40 mg/dL and greater.

Cognitive Function. Questions assessing mental cognition were asked only of respondents aged 60 or older and not to proxy respondents. These questionnaires were designed for administration in a bilingual (English/Spanish) format so that respondents could be interviewed in their preferred language. The neuropsychological measures used in the NHANES III study, were selected to assess dimensions of cognitive function typically affected in dementia. A short cognitive function index (SICF) was constructed for this analysis from these items administered at home interview and/or at a Mobile Examination Center to assess orientation, recall and attention [11,12]. A higher score indicates a higher level of cognitive function compared to a lower score. Approximate quartiles were formed. For regression analysis, persons with scores below the median were compared to those with scores above the median.

Estimates of the risk of death derive from Cox proportional hazards regression models with time to event as the time scale computed using the SURVIVAL procedure in SUDAAN. Survivors were censored at the date of the end of mortality follow-up. Logistic regression models were also done to confirm key findings.

Table 1 shows characteristics of persons aged 60+ by HDL level. In multivariate linear regression analyses, HDL was not independently associated with SICF (not shown). In proportional hazards regression models, persons with a lower compared to a higher SICF score had increased adjusted hazard of mortality (Table 2). Compared to those with HDL >40 mg/dL those with HDL of < = 40 mg/dL had increased adjusted hazard of mortality (Table 2). No significant interaction of SICF score and HDL was detected (p = 0.08). However, a significant interaction of age was seen with both SICF and HDL, the adjusted effect of both on mortality increasing with advancing age (Table 2). Table 2 shows fully adjusted hazards ratios, indicating independent associations of HDL and SICF score with survival, with strongest, significant associations among the oldest persons. Much of the association of HDL could be explained statistically by controlling for physical activity level confirming its likely causal association with both HDL level and mortality.

**Table 1:** Prevalence (%) of selected characteristics by quartile of high density lipoprotein cholesterol (HDL-C) concentration in persons aged 60y and over (N = 4911).

	HDL-C quartile				
	All	1	2	3	4
Dead	32	36	31	28	31
Cognitive function index					
<12	20	17	22	18	21
12-13	37	25	36	37	38
14-16	19	22	19	19	17
17	25	25	24	25	24
Female	57	34	53	64	79
Age 70+	49	46	50	50	51
Mexican American	2	2	3	2	2
African American	8	5	7	8	11
South region	31	31	33	32	26
Metropolitan residence	44	41	46	39	48
Unmarried	40	30	36	43	50
Education < 12 y	41	46	41	40	36
Fair-poor health	28	34	31	22	25
≥1 chronic illness	56	62	57	54	51
Mobility limitation	33	33	35	31	33
Current smoking	15	16	14	15	14
Alcohol in past month	35	29	31	37	43
No regular physician	15	15	14	14	16
No religious attendance	12	10	12	14	12
Systolic BP ≥140 mmHg	51	50	51	54	47
BMI ≥30 kg/m2	23	29	27	22	15
No physical activity	79	79	78	81	77

**Table 2:** Adjusted hazards ratios* of high density lipoprotein cholesterol, short index of cognitive function (SICF) for survival in persons aged 60 years and over.

	All	Age 60-69	Age 70-79	Age 80+
	**HR (95% CL)**			

HDL				

< = 40	1.20 (0.99-1.46)	0.92(0.67-1.27)	1.19(0.87-1.64)	1.33(1.12-1.58)**

>40	1.00	1.00	1.00	1.00

SICF				

<13.6	1.09(0.93-1.29)	1.11(081-1.53)	1.10(0.85-1.42)	1.32(1.11-1.57)**

> = 13.6	1.00	1.00	1.00	1.00

N		1947	1442	915

*adjusted for age, gender, ethnicity, education, comorbidity, region, urbanization, health status, mobility, blood pressure, smoking, alcohol, physical activity

**P < 0.01; +P < 0.05

This analysis of data from the NHANES III linked-mortality file, a nation-wide representative sample, tested for interaction of cognitive function and HDL as predictors of survival in older Americans. Both low HDL and poor cognitive function were found to independently predict poor survival chances over follow-up. However, no interaction of the two was detected. Effects increased with age. However, a previously reported association between HDL and cognitive function could not be replicated. The NHANES III provides population-based data on the association of HDL, cognitive function, and survival in a nation-wide representative sample of Americans. Oversampling of persons aged 60 and over permitted reliable estimates for this group. However, several unavoidable limitations of the present study include possible bias arising from survey non-response and missing values for some variables and from possible changes in HDL and cognitive function and/or other variables over the follow-up period. Adjusting for age, HDL and SICF were also independently associated with death in logistic regression models (p < 0.01) with no significant interaction between them (p = 0.48). Further longitudinal studies with measures of HDL subtypes, apolipoprotein A-1, apoE, and multiple dimensions of cognition and Alzheimer's disease biomarkers would be helpful.

## List of abbreviations

HDL: high density lipoprotein cholesterol; NHANES III: Third National Health and Nutrition Examination Survey; SICF: short index of cognitive function

## Competing interests

The authors declare that they have no competing interests.

## Authors' contributions

Both authors made substantial contributions to the conception, design, data analysis, drafting and revision of the manuscript. Both authors read and approved the final manuscript.

## Authors' information

TO is Professor of Medicine, Howard University College of Medicine and Chief of the Division of Geriatrics, Howard University Hospital. RG is Adjunct Professor of Medicine, Howard University College of Medicine.
